# Dissemination of Early Intervention Program for Preschool Children on the Autism Spectrum into Community Settings: An Evaluation

**DOI:** 10.3390/ijerph19052555

**Published:** 2022-02-23

**Authors:** Anne Masi, Syeda Ishra Azim, Feroza Khan, Lisa Karlov, Valsamma Eapen

**Affiliations:** 1 School of Psychiatry, Faculty of Medicine, University of New South Wales, Sydney 2052, Australia; a.masi@unsw.edu.au (A.M.); syeda_ishra.azim@unsw.edu.au (S.I.A.); feroza.khan@unsw.edu.au (F.K.); lisa.karlov@health.nsw.gov.au (L.K.); 2 South Western Sydney Local Health District, Liverpool 2170, Australia; 3 Academic Unit of Child Psychiatry, Level 1, Mental Health Centre, Liverpool Hospital, 1 Elizabeth Street, Liverpool 2170, Australia; 4 Ingham Institute for Applied Medical Research, Liverpool 2170, Australia

**Keywords:** autism spectrum disorder, early learning centre, Early Start Denver Model, intervention, change, early childhood

## Abstract

We aimed to address a policy-relevant research area with high priority, namely disseminating early intervention for children on the autism spectrum into mainstream community settings. The study cohort comprised 47 children with a diagnosis of Autism Spectrum Disorder (ASD) receiving the Early Start Denver Model (ESDM) intervention: 23 children attending an Autism Specific Early Learning and Care Centre (ASELCC) and 24 children attending a mainstream preschool setting. Group comparisons revealed that the overall response to intervention was in the majority of cases not significantly different between settings. One difference was found in that children in the mainstream preschool setting showed a significant reduction in externalising behaviours compared to the children attending the autism-specific preschool. Intervention duration was found to influence outcomes with a one-month increase in duration found to improve expressive language skills. While the results need to be interpreted with caution due to the small sample size, these findings suggest that early intervention can be successfully delivered in both autism-specific and mainstream settings. However, those families needing additional parent support may be better served by a specialised service.

## 1. Introduction

The prevalence of Autism Spectrum Disorder (ASD) is increasing, with the rate progressing from 1 in 2500 people 40 years ago to the current prevalence rate estimate of 1 in 54 children in the United States in 2016 [1]. Australia has one of the highest reported prevalence rates in the world, with the rate increasing from a parent-reported adjusted prevalence estimate of ASD diagnosis among 10- to 11-year-olds of 2.4% (95% CI 1.6 to 2.9) for those born in 1999/2000 to 3.9% (95% CI 3.2 to 4.5) for those born in 2003/2004 [2,3].

Underpinned by a genetic vulnerability, ASD has been proposed to arise from a developmental cascade effect whereby a deficit in attention to social stimuli leads to impaired interactions with primary caregivers, thereby affecting the social communication domain of development [4]. This in turn results in differences and deviations in the development of the neuronal circuitry responsible for social cognition and language development, with consequent adverse impacts on later behavioural and functional domains dependent on these early processes [5]. This model suggests the importance of early intervention for ASD and is supported by studies showing that the earlier the intervention, the better outcomes [6,7]. Early intervention is ideal as this can take advantage of brain plasticity in the early preschool years, enabling the establishment and reorganisation of neuronal networks in response to environmental stimulation [5]. Recent meta-analyses have shown that Early Intensive Intervention (EII) is the treatment of choice for young children with ASD [8], and superior outcomes have been observed with entry into EII at the earliest possible age [9,10]. The Early Start Denver Model (ESDM) [11] is a manualised, comprehensive play-based intervention that integrates Applied Behavior Analysis (ABA) with relationship-based Pivot Response Treatment (PRT) and developmental approaches. In Australia, community-based studies of ESDM have been conducted in long day care settings at Autism Specific Early Learning and Care Centres (ASELCCs) established specifically for children with ASD through funding by the Australian Commonwealth Government. While these interventions implemented through group delivery have been shown to be effective in this context [12,13], there are no studies to date that directly compare outcomes of early intervention for children with ASD provided within autism-specific services to those provided in mainstream preschool settings using the same intervention model, ESDM.

The project was linked to an existing program, the Child and Family Outcome Study (CFOS), which was set up in 2010 and funded by the Department of Social Services (DSS), Commonwealth Government of Australia. It utilised a ‘hub and spoke’ model to compare ESDM intervention provided by the KU Children’s Services ASELCC, a specialised service, and the same program provided within three KU Children’s Services mainstream preschools in Sydney, New South Wales (NSW), Australia (KU Dissemination sites—KUD), for children with an ASD diagnosis. It aimed to examine any differences in response to receiving the ESDM intervention in the autism-specific setting versus the mainstream preschool setting. This is expected to assist in determining best practices for the delivery of early intervention services and will provide a much needed evidence base for informing policy development for early intervention for preschool children with ASD in the Australian context.

## 2. Materials and Methods

### 2.1. Participants and Sample

Twenty-four children receiving ESDM at the three mainstream preschools (Briar Park, Cobbitty and Macquarie Fields—KUD sites) were recruited when they started at their respective preschools. Using a propensity score matching procedure [14], 23 children from the ASELCC who received the ESDM in a specialised setting were matched. The selection was made from 140 children in the NSW ASELCC dataset. Participants who had no missing data in Mullen Scales of Early Learning (MSEL) and the Autism Diagnostic Observation Schedule-2 (ADOS-2) measurement in both baseline and exit assessments (*n* = 85) were eligible to be matched with the 24 children from the KUD sites in order to maximise the size of the dataset. The samples were matched on sex, age at ADOS-2 assessment, and ADOS-2 Calibrity Severity Score with a match tolerance of 0.1.

The resulting sample consisted of 23 children from the ASELCC and 24 children from KUD sites: Briar Cottage (11), Cobbitty (5) and Macquarie Fields (8). All participating children had a DSM-5 diagnosis of Autistic Disorder or Autism. The average age of the children attending the ASELCC at enrolment was 50 months (SD 7.0 months, range: 34 months–64 months), and the average age of the children attending the KUD sites at the date of the baseline ADOS-2 assessment was 52 months (SD 5.4 months, range: 37 months to 60 months) (see Table 1). The gender ratio was comparable for the two settings (ASELCC 78.3% male; KUD 66.7% male). Over three-quarters of the children from the ASELCC came from a culturally and linguistically diverse background (CALD), whilst only 40% of the children from the KUD preschools with complete data provided came from a CALD background. Overall, the return rate for demographic questionnaires was much higher at the ASELCC (95.7%) compared to the KUD (41.7%). This low return rate at KUD, and high no response rates at both the ASELCC and KUD, meant that the impact that family factors had on child outcomes, specific to the setting in which the intervention was delivered, could not be fully investigated.

### 2.2. Measures

Measures were administered and questionnaires were completed at baseline (entry) and at follow up—post ESDM intervention—for both groups of children. Follow-up data were collected either when the child exited the service or at the end of the academic year, which equated to approximately ten months after baseline data were collected. Measures administered at both time points were the measures already in use for the ASELCC entry and follow-up assessments [15]. The assessments were completed within the ASELCC (as part of the scheduled exit and entry assessments) and within the KUD mainstream preschools where the participating child attended. Table 2 provides a summary of the instruments and a guide to interpretation.

#### 2.2.1. Autism Traits

The Autism Diagnostic Observation Schedule-2nd Edition (ADOS-2) [16] is a semi-structured, standardised diagnostic observational assessment used to confirm the DSM-5 [17] diagnosis and determine the severity of ASD. The ADOS-2 module, administered by the trained clinician or researcher, is determined by the child’s age and expressive language ability. Module 1 was administered to children aged 31 months and older who had no speech or only single words/simple phrases, Module 2 to children with phrase speech who were not verbally fluent and Module 3 to children with fluent language. The time between ADOS-2 assessments at the ASELCC (9.81 months, SD 2.27) and at the KUD (9.02 months, SD 1.51 months) was not significantly different.

To assess further for traits of autism, a screening measure was completed by parents or caregivers. The Social Communication Questionnaire (SCQ) [18] is a 40-item (yes/no) parent-report screening measure that evaluates communication, reciprocal social interaction, and restricted and repetitive behaviours and interests which are core diagnostic criteria for ASD [17]. This questionnaire was included in the study to provide reliable screening for ASD and as a measure of change over time.

The Repetitive Behavior Scale—Revised (RBS-R) [19] was completed by parents or caregivers to measure the extent of repetitive behaviours. The RBS-R is a 44-item self-report questionnaire and provides a quantitative, continuous measure of the full spectrum of repetitive behaviours in ASD. The RBS-R consists of six subscales, including Stereotyped Behavior, Self-injurious Behavior, Compulsive Behavior, Routine Behavior, Sameness Behavior and Restricted Behavior.

#### 2.2.2. Child Development Skills

At both time points, a developmental assessment was conducted using the Mullen Scales of Early Learning (MSEL) [20]. The MSEL is a researcher-administered assessment that was used to evaluate cognitive and language function. The MSEL assesses development across key domains, including receptive and expressive language, perceptual abilities and fine motor skills. For each domain, raw scores and a corresponding age equivalence score were obtained. A standardised development quotient (DQ) was subsequently calculated by dividing the age-equivalent score on each of the MSEL subscales with the child’s chronological age and then multiplying by 100.

#### 2.2.3. Adaptive Functioning

The Vineland Adaptive Behavior Scales Second Edition (VABS-II) [21] (caregiver self-report form) is a standardised, norm-referenced evaluation tool for children where parents report on the child’s adaptive/functional skill level in the domains of Communication, Daily Living Skills, Socialisation and Motor Skills, which form the Adaptive Behavior Composite. Additionally, the Maladaptive Behavior Index is a composite of Externalising and Internalising Behaviors.

#### 2.2.4. Child Behaviour

The Child Behavior Checklist for Ages 1.5-5 (CBCL) [22] is a parent report form used to screen for emotional, behavioural and social problems. The preschool checklist contains 100 problem behaviour questions. Parents or caregivers rate the child’s behaviour on a 3-point scale (not true, somewhat or sometimes true, and very true or often true), as it occurs now, or within the previous two months, to accommodate the rapid development and behavioural changes which are common in the preschool age range. There are seven empirically based syndrome scales: Aggressive Behavior, Anxious/Depressed, Attention Problems, Emotionally Reactive, Sleep Problems, Somatic Complaints and Withdrawn. The Aggressive Behavior, Anxious/Depressed and Attention Problems scales form the Dysregulation Profile. There are also two broad groupings of syndromes: Internalising, consisting of the Anxious/Depressed, Emotionally Reactive, Somatic Complaints and Withdrawn scales, and Externalising, consisting of the Aggressive Behavior and Attention Problems scales. A Caregiver-Teacher Report Form (C-TRF) version is also completed by the child’s teacher or keyworker at the ASELCC and KUD sites.

The Short Sensory Profile 2 (SSP-2) [23] is a 34-item parent questionnaire designed to measure behaviours associated with abnormal responses to sensory stimuli. The SSP-2 provides scores in four quadrants based on the child’s threshold to sensory input and their method of self-regulation. The four quadrants are Seeking, Avoiding, Sensitivity and Registration.

#### 2.2.5. Parental Stress and Quality of Life

The Quality of Life in Autism (QoLA) [24] questionnaire is also completed by the parents or caregivers. It is a 48-item questionnaire assessing two subscales: Parents’ overall perception of their quality of life and parents’ perception of the degree to which their child’s autism symptoms are problematic. The Parenting Stress Index-4 Short Form (PSI-4 SF) [25] consists of 36 items measuring Parental Distress, Parent–child Dysfunctional Interaction and Difficult Child domains and is completed by the parents.

#### 2.2.6. Sociodemographic

A family history questionnaire consisting of sociodemographic information as well as associated issues of relevance to the child and the family is also completed by the parent or caregiver at entry into the intervention program.

### 2.3. Procedure

Staff were trained in the ESDM by a certified ESDM training facilitator. All staff members who were trained in the delivery of the ESDM within both the ASELCC and mainstream services received the same number of hours of training. The implementation of the ESDM was the same across the two settings, ensuring fidelity of the intervention program. Within KU Children’s services, the staff ratio required for children with ASD in the mainstream preschool settings is 1 staff member for 3 children with ASD, which offered comparable staff to child ratios for the ESDM program delivered at the ASELCC and as part of the mainstream preschool program. The burden on stakeholders was reduced by utilising the entry and exit assessments already completed for children attending the ASELCC. The study was conducted in accordance with the Declaration of Helsinki and approved by the Human Research Ethics Committee of the University of New South Wales (HREC Approval No: HC14267 dated 30 November 2016).

### 2.4. Statistical Analysis

Descriptive statistics were used to show sociodemographic and clinical characteristics of participants at baseline. Student *t*-tests were conducted to compare the child’s pre- and post-intervention outcome between the ASELCC and the KUD settings. Baseline scores between the settings were compared by a series of *t*-tests. Pre- to post-intervention change scores were calculated by subtracting the pre-intervention score from the post-intervention score for each child. Multiple regression was used to investigate whether the treatment response differed by the setting—autism-specific (ASELECC) or integrated with mainstream (KUD). In the regression model, the setting was used as a predictor of treatment response, controlling for the duration in the study and sex. As age was already matched, it was not included in the models. Statistical analysis was carried out using SPSS v. 26 [26] and the R version 4.0.3 [27]. Only Carer 1 data could be used to investigate family functioning as questionnaire return rates for Carer 2 were very low.

## 3. Results

Mean scores on all assessments and parent completed questionnaires were compared between children attending the ASELCC and those attending KUD settings at baseline and again at follow up (Table 3). At baseline, ASELCC participants had more communication problems measured by SCQ (*p* = 0.02) than KUD participants. KUD participants had higher externalising (*p* < 0.01) and internalising behaviour (*p* < 0.01) problems as measured by the Caregiver Teacher Report Form in the questionnaires completed at baseline. Participants at KUD sites also had higher internalising behaviour problems than participants at the ASELCC in the same questionnaire completed at follow up (*p* < 0.01). Finally, the children attending the KUD sites had higher scores on the Sensory Seeking quadrant of the Short Sensory Profile than the ASELCC children at baseline (*p* = 0.03).

Multiple regression was used to investigate whether the treatment response differed based on whether the child attended the ASELCC or the KUD (Table 4). The setting was used as a predictor of treatment response, controlling for the duration of the study and sex, as age was already matched. It was found that children who attended the KUD preschools showed significant reduction in externalising behaviours as measured by the VABS-II compared to the children attending the ASELCC (β = 2.85, *p* = 0.03). Though setting did not contribute significantly to response to intervention in any other measure (Table 4), sex was significantly associated with receptive language (MSEL) and motor skills (VABS-II: fine and gross motor skills); male participants made greater improvements in receptive language than female participants (β = −9.07, *p* = 0.04) and also in fine motor skill development (β = −11.29, *p* = 0.02), both as measured by the MSEL. In contrast, females showed marginally greater improvements in motor skills (fine and gross motor) as measured by the VABS-II (β = 0.85, *p* = 0.02). Duration was also seen to contribute to response to intervention for expressive language, where a one=month increase in duration resulted in improved expressive language skills (β = 2.93, *p* = 0.02). Again, results need to be interpreted with caution as the sample size is small and sample sizes at follow up are substantially lower than at baseline.

## 4. Discussion

In comparing the two settings for delivery of the ESDM—an autism-specific preschool (ASELCC) and embedding of the program within mainstream preschools (KUD)—it was found that similar gains were made by the children across the two settings. Broadly speaking, then, ESDM appears to be as effective if conducted in an autism-specific preschool as in a mainstream preschool.

One significant difference between settings did arise. The regression analyses identified that children in the mainstream preschool setting showed a significant reduction in externalising behaviours captured by the VABS-II compared to the children attending the autism-specific preschool. This difference could indicate a real difference in treatment experience for children based on where that treatment occurred. Alternatively, it could reflect sampling artefacts, particularly seeing as at baseline the children attending the mainstream preschool setting had on average greater levels of externalising and sensory seeking; the children in the mainstream setting therefore had a greater potential for improvement, having started from a lower point. A replication of this study in which participants were matched on all baseline measures could shed further light on whether the significant differences found here reflect real differences between settings or sampling factors.

In the mainstream preschool setting in which this study was conducted, the inclusiveness was made possible through government funding provided against each named child for early intervention and support through the National Disability Insurance Scheme. This facilitated higher child-to-staff ratios as well as training for the mainstream preschool staff in early intervention for autism. Given that both settings are resourced through the government in Australia, further research should focus on better understanding the reasons behind the choice that parents make in terms of the child’s placement in an autism-specific centre versus the mainstream setting. There may be factors related to the parent or the child that drive this initial decision, which may also be relevant for predicting treatment outcomes for children. In this way, differences between settings in terms of child outcomes may reflect parental choices prior to the beginning of treatment as well as the child’s experience within the treatment settings.

The current study was conducted with several limitations. The sample size was small, reflecting the small population of families receiving treatment via ESDM in early education settings in Sydney, Australia. Compounding this, the return rate of parent completed questionnaires was low for children attending mainstream preschool settings. However, the current study has addressed an important question using gold-standard assessment and has significant clinical implications. It is important to demonstrate that early intervention for autism can be effective regardless of setting. It will be equally important to consider whether families’ selection of setting is made in reference to currently unknown factors that can then affect a child’s response to treatment. Additionally, examination of longitudinal outcomes for children attending the two treatment settings would be beneficial, perhaps utilising data linkage with health, education and social services data, in order to have a comprehensive understanding of the long-term benefits of early intervention for autism in the Australian setting.

## 5. Conclusions

Early intervention for autism can be successfully delivered in autism-specific intervention settings and in mainstream settings provided that staff training on intervention programs and staff-to-child ratios and related requirements can be put in place. The behavioural profile of the child and the need for parental support should be a key consideration while decisions are being made about the placement setting as those with more severe autism symptoms and behavioural difficulties may need additional child and parent supports that may not be feasible in mainstream settings. Future research should examine parental preferences, attitudes and expectations about choice of early intervention and educational placement setting and potentially utilise a longitudinal design to understand longer term outcomes for children receiving early intervention for autism in Australia.

## Figures and Tables

**Table 1:** Participant demographic statistics.

	KUD		ASELCC		*p*-Value
	*n*/Mean	%/sd	*n*/Mean	%/sd	
Child age	4.31	0.45	4.19	0.62	0.45
Child sex					
Male	16	66.67	18	78.26	0.37
Female	8	33.33	5	21.74	
Child birth order					
First	6	60.00	15	68.18	0.81
Second	3	30.00	4	18.18	
Third or higher	1	10.00	3	13.60	
^1^ CALD					
No	6	60.00	5	23.81	0.11
Yes	4	40.00	16	76.19	
Parent Education Carer 1					
Secondary or less	1	12.50	7	33.33	0.50
Tertiary	5	62.50	9	42.86	
Postgraduate	2	25.00	5	23.81	
Parent Occupation Carer 1					
Employed (including leave)	1	16.67	9	47.37	0.58
Home duties (including caring)	3	50.00	7	36.84	
Unemployed/retired	2	33.33	3	15.78	
Parent Income Carer 1					
Less than $40,000	2	25.00	6	33.33	0.63
$40,000–$100,000	4	50.00	10	55.56	
Over $100,000	2	25.00	2	11.11	

^1^ CALD = Culturally and Linguistically Diverse.

**Table 2:** Meaning of higher scores and change score direction for each measure.

Measure	Higher Score	Change Score Direction Indicating Improvement
Autism Diagnostic Observation Schedule—2nd Edition	More signs of autism	Negative
Social Communication Questionnaire	More abnormal behaviour	Negative
Repetitive Behavior Scale—Revised	More problematic behaviour	Negative
Mullen Scales of Early Learning	Better skills	Positive
Vineland Adaptive Behavior Scales—II Adaptive Behavior and Domains	Better function	Positive
Vineland Adaptive Behavior Scales—II Maladaptive Behavior Index	Worse function	Negative
Child Behavior Checklist	More problematic behaviour	Negative
Quality of Life in Autism Part A	Greater quality of life	Positive
Quality of Life in Autism Part B	Fewer problematic behaviours	Positive
Parental Stress Index	More stress	Negative

**Table 3:** Mean scores at baseline and follow up.

Measure	Baseline							Follow Up						
	KUD			ASELCC			*p*	KUD			ASELCC			*p*
	n	Mean	SD	n	Mean	SD		n	Mean	SD	n	Mean	SD	
Autism Traits														
ADOS-2														
Calibrated Severity Score	24	6.58	1.56	23	6.43	1.73	0.76	22	6.14	1.93	23	6.78	1.70	0.24
Social Affect	24	12.75	3.38	23	13.39	4.64	0.59	22	10.86	4.09	23	12.96	3.78	0.08
Restricted Repetitive Behavior	24	3.54	1.53	23	3.91	2.15	0.50	22	3.86	1.96	23	4.83	2.06	0.12
SCQ														
Social	18	6.39	2.64	18	7.94	3.47	0.14	11	5.55	3.59	16	7.56	4.03	0.19
Communication	19	5.85	2.51	17	8.51	3.93	0.02	11	5.25	3.08	15	8.04	4.24	0.06
Repetitive Measure	17	4.71	2.46	17	4.17	2.47	0.52	11	4.61	2.33	16	4.64	2.34	0.97
Total	16	17.61	6.55	16	20.49	7.33	0.25	11	16.28	5.31	15	21.7	8.76	0.06
RBS-R														
Stereotyped	19	8.79	6.67	17	7.76	3.73	0.57	10	5.90	5.04	16	6.81	4.17	0.64
Self-Injurious	19	3.68	5.72	17	3.00	2.96	0.65	10	4.80	6.20	16	2.19	3.73	0.25
Compulsive	19	3.79	3.74	17	3.00	2.12	0.44	10	4.30	4.76	16	3.62	3.1	0.70
Ritualistic and Same	19	7.53	8.55	17	4.65	4.26	0.21	10	6.80	5.79	16	8.50	8.69	0.56
Restricted	19	4.32	2.98	17	3.18	2.40	0.21	10	4.10	2.60	16	3.19	2.79	0.41
Total	19	28.11	24.59	17	21.59	10.18	0.30	10	25.9	20.05	16	24.31	17.18	0.84
Developmental Skills														
MSEL DQ														
Visual Reception	22	56.58	23.03	23	43.87	19.14	0.05	23	66.2	32.32	21	49.68	28.04	0.08
Fine Motor	23	57.22	18.69	23	49.58	18.86	0.17	23	59.73	20.13	22	51.66	23.46	0.22
Receptive Language	24	46.35	27.81	23	32.37	23.34	0.07	23	53.25	29.5	22	38.49	26.45	0.08
Expressive Language	24	46.06	24.97	23	33.27	24.01	0.08	23	51.78	28.24	23	37.07	24.79	0.07
Adaptive functioning														
VABS-II														
ABC SS	12	64.83	9.20	14	61.79	16.05	0.55	8	71.88	13.57	13	63.46	15.09	0.20
Maladaptive	16	19.88	2.73	17	18.88	2.29	0.27	9	19.00	2.45	16	18.56	3.08	0.70
Internalising	16	20.94	1.95	17	19.65	1.93	0.07	9	20.00	2.50	16	19.44	2.78	0.61
Externalising	16	16.94	3.13	17	16.53	2.65	0.69	9	17.11	3.30	16	16.25	3.00	0.53
Child Behavior														
CBCL														
Dysregulation Profile	14	184.14	28.88	16	174.31	12.56	0.25	10	177	28.17	11	176.36	16.95	0.95
Externalising	14	59.21	14.85	16	55.69	8.81	0.45	10	56.9	13.58	11	57.45	9.82	0.92
Internalising	11	67.64	9.12	6	66.67	8.07	0.83	10	61.1	10.47	10	61.90	10.45	0.87
CTRF														
Externalising	17	70.76	8.45	13	59.62	4.41	<0.01	21	67.43	10.13	9	58.44	11.18	0.06
Internalising	18	76.00	10.56	19	59.68	7.86	<0.01	18	75.28	9.30	21	61.81	10.69	<0.01
SSP-2														
Seeking	21	19.14	6.09	13	14.92	4.84	0.03	9	18.89	9.31	16	15.56	5.15	0.34
Avoiding	21	27.10	9.12	13	22.46	8.29	0.14	9	26.33	9.53	16	23.88	8.84	0.53
Sensitivity	21	29.95	9.25	13	29.54	7.89	0.89	9	29.67	12.03	16	28.62	8.86	0.82
Registration	21	18.29	7.84	13	17.62	6.76	0.79	9	17.78	10.85	16	16.19	6.84	0.70
Family Functioning														
QoLA Carer 1														
Part A	20	98.45	19.66	18	104.06	28.29	0.49	11	103.18	21.08	17	108.47	21.32	0.53
Part B	20	68.75	19.80	18	78.94	16.63	0.09	11	69.45	18.91	17	83.24	17.33	0.07
PSI Carer 1														
Parental Distress	21	32.67	10.48	18	30.22	12.24	0.51	11	32.27	10.15	15	29.27	8.66	0.44
Parent–Child Dysfunctional Interaction	21	31.33	9.31	18	30.44	7.49	0.74	11	31.09	7.42	15	27.27	6.66	0.19
Difficult Child	21	33.00	8.93	17	32.71	9.73	0.92	11	34.82	8.01	15	31.00	8.96	0.27

NOTE: ABC SS—Adaptive Behavior Composite Standard Score; ADOS-2 = Autism Diagnostic Observation Schedule—2nd Edition; CBCL = Child Behavior Checklist; CTRF = Child Teacher Rating Form; DQ = Development Quotient; MSEL = Mullen Scales of Early Leaning; PSI = Parental Stress Index; QoLA = Quality of Life in Autism; RBS-R = Repetitive Behavior Scales—Revised; SCQ = Social Communication Questionnaire; SSP-2 = Short Sensory Profile, 2nd Edition; VABS-II = Vineland Adaptive Behavior Scales—2nd Edition.

**Table 4:** Predictors of change in the severity of autism symptoms, child development skills, adaptive functioning and child behavior.

Autism Traits																
**ADOS-2**	**Social Affect**		**RRB**				**Severity Score**									
	**β**	***p***	**β**		***p***		**β**		***p***							
Site (KUD = 1)	−1.70	0.12	−0.85		0.23		−0.85		0.19							
Duration	−0.07	0.81	0.08		0.64		0.04		0.83							.
Sex (Female = 1)	−0.47	0.70	0.52		0.51		0.07		0.92							
**SCQ**	**Communication**		**Restricted Social Interaction**				**Repetitive Behaviour**			**SCQ Total**						
	**β**	***p***	**Β**		***p***		**β**		***p***	**β**	***p***					
Site (KUD = 1)	1.10	0.45	−2.63		0.23		0.03		0.98	−3.67	0.14					
Duration	0.22	0.57	−0.39		0.43		0.04		0.89	0.05	0.94					
Sex (Female = 1)	0.80	0.60	1.21		0.54		−0.16		0.90	1.74	0.46					
**RBS-R**	**Stereotyped**		**Self Injurious**				**Compulsive**			**Ritualistic and Same**		**Restricted**			**Total**	
	**β**	***p***	**β**		***p***		**Β**		***p***	**β**	***p***	**β**	***p***		**β**	***p***
Site (KUD = 1)	−3.95	0.12	1.08		0.39		−0.47		0.75	−3.86	0.14	−0.74	0.27		−7.93	0.14
Duration	−0.47	0.49	−0.02		0.96		0.23		0.58	0.13	0.86	−0.28	0.13		−0.42	0.78
Sex (Female = 1)	2.74	0.27	0.82		0.51		1.72		0.25	−0.80	0.75	0.28	0.67		4.76	0.38
**Development Skills**																
**MSEL**	**Visual Reception**		**Fine Motor**				**Receptive Language**			**Expressive Language**						
	**β**	***p***	**β**		***p***		**β**		***p***	**β**	***p***					
Site (KUD = 1)	9.54	0.10	2.18		0.60		2.39		0.54	4.33	0.23					
Duration	−0.32	0.86	−0.22		0.87		0.17		0.89	2.93	0.02					
Sex (Female = 1)	−10.09	0.13	−11.29		0.02		−9.07		0.04	−5.56	0.16					
**Adaptive Functioning**																
**VABS-II**	**Communication**		**Daily Living Skills**		**Socialisation**		**Motor Skills**		**Internalising**		**Externalising**		**Maladaptive**		**Adaptive Behaviour**	
	**β**	***p***	**β**	***p***	**Β**	***p***	**β**	***p***	**β**	***p***	**β**	***p***	**β**	***p***	**β**	***p***
Site (KUD = 1)	0.03	0.92	−0.07	0.81	0.11	0.73	0.01	0.97	0.16	0.91	2.85	0.03	1.75	0.12	6.49	0.24
Duration	−0.13	0.70	−0.11	0.73	−0.02	0.95	0.05	0.85	0.18	0.59	0.47	0.12	0.47	0.08	2.09	0.12
Sex (Female = 1)	0.40	0.25	0.48	0.18	0.65	0.06	0.85	0.02	0.75	0.56	0.18	0.87	−0.17	0.87	4.16	0.44
**Child Behaviour**																
**CBCL-*p***	**Internalising**		**Externalising**		**Dysregulated Profile**											
	**β**	***p***	**β**	***p***	**β**	***p***										
Site (KUD = 1)	3.53	0.53	1.58	0.73	−4.30	0.62										
Duration	−2.21	0.14	−0.63	0.65	−3.12	0.25										
Sex (Female = 1)	1.60	0.73	0.70	0.85	0.79	0.91										
**CTRF**	**Internalising**		**Externalising**													
	**β**	***p***	**β**	***p***												
Site (KUD = 1)	−1.7	0.56	−0.47	0.92												
Duration	−0.87	0.42	−0.23	0.79												
Sex (Female = 1)	−1.06	0.75	−3.29	0.31												

NOTE: ADOS-2 = Autism Diagnostic Observation Schedule—2nd Edition; CBCL = Child Behaviour Checklist; CTRF = Child Teacher Rating Form; DQ = Development Quotient; KUD—KU Dissemination site; MSEL = Mullen Scales of Early Leaning; RBS-R = Repetitive Behaviour Scales - Revised; RRB = Restricted Repetitive Behaviour; SCQ = Social Communication Questionnaire; VABS-II = Vineland Adaptive Behaviour Scales—2nd Edition.

## Data Availability

Data will be made available to those who require it upon request to the corresponding author.
